# Risk Factors for Inadequate TB Case Finding in Rural Western Kenya: A Comparison of Actively and Passively Identified TB Patients

**DOI:** 10.1371/journal.pone.0061162

**Published:** 2013-04-25

**Authors:** Anna H. van’t Hoog, Barbara J. Marston, John G. Ayisi, Janet A. Agaya, Odylia Muhenje, Lazarus O. Odeny, John Hongo, Kayla F. Laserson, Martien W. Borgdorff

**Affiliations:** 1 University of Amsterdam, Academic Medical Centre, Amsterdam, The Netherlands; 2 Kenya Medical Research Institute (KEMRI), KEMRI/Centers for Disease Control (CDC) Research and Public Health Collaboration, Kisumu, Kenya; 3 United States Centers for Disease Control and Prevention (CDC), Division of Global AIDS, Atlanta, Georgia, United States of America; 4 Kenya Medical Research Institute (KEMRI) Centre for Global Health Research, Kisumu, Kenya; 5 United States Centers for Disease Control and Prevention (CDC), Global AIDS Program, Nairobi, Kenya; 6 Public Health Service, Department of Infectious Diseases, Amsterdam, The Netherlands; University of Ottawa, Canada

## Abstract

**Background:**

The findings of a prevalence survey conducted in western Kenya, in a population with 14.9% HIV prevalence suggested inadequate case finding. We found a high burden of infectious and largely undiagnosed pulmonary tuberculosis (PTB), that a quarter of the prevalent cases had not yet sought care, and a low case detection rate.

**Objective and methods:**

We aimed to identify factors associated with inadequate case finding among adults with PTB in this population by comparing characteristics of 194 PTB patients diagnosed in a health facility after self-report, *i.e.,* through passive case detection, with 88 patients identified through active case detection during the prevalence survey. We examined associations between method of case detection and patient characteristics, including HIV-status, socio-demographic variables and disease severity in univariable and multivariable logistic regression analyses.

**Findings:**

HIV-infection was associated with faster passive case detection in univariable analysis (crude OR 3.5, 95% confidence interval (CI) 2.0–5.9), but in multivariable logistic regression this was largely explained by the presence of cough, illness and clinically diagnosed smear-negative TB (adjusted OR (aOR) HIV 1.8, 95% CI 0.85–3.7). Among the HIV-uninfected passive case detection was less successful in older patients aOR 0.76, 95%CI 0.60–0.97 per 10 years increase), and women (aOR 0.27, 95%CI 0.10–0.73). Reported current or past alcohol use reduced passive case detection in both groups (0.42, 95% CI 0.23–0.79). Among smear-positive patients median durations of cough were 4.0 and 6.9 months in HIV-infected and uninfected patients, respectively.

**Conclusion:**

HIV-uninfected patients with infectious TB who were older, female, relatively less ill, or had a cough of a shorter duration were less likely found through passive case detection. In addition to intensified case finding in HIV-infected persons, increasing the suspicion of TB among HIV-uninfected women and the elderly are needed to improve TB case detection in Kenya.

## Introduction

Prompt case finding is an important pillar of global tuberculosis (TB) control [1]. The 5.8 million TB cases that were notified globally in 2009 represented only 63% of the estimated number of new TB cases, and case detection was lower in the African region [2]. TB case finding in countries with a high TB-burden but low income is mostly passive and relies on self-reporting of symptomatic persons to the health service. Delays in diagnosis through passive case detection have been associated with patient- and provider-related factors [3], [4]. Most studies on case finding have investigated risk factors associated with delay in diagnosis of TB patients found through passive case detection [3], [4]. Few studies have compared TB patients found through passive case detection with those identified through prevalence surveys or other active case finding efforts. These studies were in populations with low HIV prevalence [5]–[8], had small sample sizes [6] or were restricted to household contacts only [9], [10].

We previously conducted a TB prevalence survey in a rural area in western Kenya with high HIV prevalence and found a high burden of undiagnosed pulmonary tuberculosis (PTB), and a need to improve case finding. The prevalence of bacteriologically-confirmed PTB was 6.0 per 1000 (95% confidence interval (CI) 4.6–7.4), and of smear-positive TB 2.5 per 1000 (95%CI 1.6–3.4). Of the identified cases, 95% were not on TB treatment at the time of survey [11]. We estimated the case detection rate, especially that of HIV-infected TB-cases, to be below the WHO target of 70% [12].

To inform the development of strategies that could improve TB case finding in this population, we assessed factors affecting TB case finding by comparing characteristics of patients with PTB diagnosed at health facilities through passive case detection with characteristics of PTB patients identified through active case finding during the prevalence survey.

## Methods

### Ethical Approval

The protocols for the study on care seeking in passively detected TB cases and the prevalence survey were approved by the Kenya Medical Research Institute Scientific Steering Committee and Ethics Review Committee and by the US Centers for Disease Control and Prevention Institutional Review Board (IRB-G). Written informed consent was obtained of the participants.

### Study Population

All study participants resided in the Asembo (Rarieda District), and Gem District areas in Nyanza Province, western Kenya. These rural areas, with a population density of 270 person per km^2^, are included in a health and demographic surveillance system (HDSS) [13]. In the Nyanza province in 2007, the TB notification rate was 431/100,000 [14], and HIV prevalence was 14.9% in those aged 15–64 years [15]. TB control was supervised by the division of leprosy, tuberculosis and lung diseases (DLTLD) of the ministry of health, and the area had approximately 2.5 TB diagnostic and 7.8 TB treatment facilities per 100,000 population [16].

### Patients Identified through Passive Case Detection

Between October 2007 and September 2008, all persons of 18 years and older who resided in the HDSS area, started treatment for PTB after self-reporting with TB symptoms to health facilities serving the HDSS population, and had not received TB treatment in the last 2 years, were eligible for a study on care seeking. Patients were enrolled consecutively at the TB clinics until the intended sample size (of 400 self-reported and prevalent cases combined) was reached. TB diagnosis followed the national DLTLD guidelines [17]: patients suspected of having TB were investigated by direct Ziehl-Neelsen (ZN) sputum smear microscopy. If smears were negative, a clinical diagnosis was made by a clinical or medical officer, aided by chest radiography and/or lack of response to a course of broad-spectrum antibiotics. Diagnostic mycobacterial cultures were not available. The patients were interviewed as soon as possible, within 4 weeks after TB treatment initiation, by trained study nurses who used a structured pre-tested questionnaire that was developed based on prior in-depth interviews [18].

### Patients Identified through Active Case Detection

A TB prevalence survey was conducted between August 2006 and December 2007 in the Asembo and Gem areas and included 20,566 residents aged 15 years and above from 40 randomly selected clusters, that together included approximately 38% of the villages in the area. The detailed methods have been described previously [11]. Briefly, all participants were examined with a symptoms questionnaire, chest radiograph and two sputum samples for microscopic examination. Participants in whom TB was suspected based on either symptoms, CXR abnormalities, or sputum microscopy provided an additional sputum sample for mycobacterial culture. We identified 123 persons with bacteriologically confirmed PTB, defined as either one culture sample positive for *M. tuberculosis*, or two sputum smears positive for acid fast bacilli not explained by isolation of non-tuberculous mycobacteria. Of those, 117 were not on TB treatment at the time of survey, and are hereafter referred to as patients identified though active case detection or prevalent cases. These patients were referred to health facilities in the area, initiated on anti-tuberculosis treatment, and interviewed about care-seeking by the same study nurses as the passively detected patients. Patients who had moved out of the area were traced if possible.

For consistency, we included patients identified through passive case detection in this analysis only if they resided in the Asembo and Gem HDSS areas, and prevalent patients only if they were aged 18 years or older, and had completed a care-seeking interview. Data were collected on socio-demographic information, presence and duration of symptoms, self-reported ability to work or do normal chores at the time of TB diagnosis or participation in the prevalence survey, and contact with formal and informal health care providers. All patients were offered provider-initiated HIV counselling in accordance with national policy, and after consenting, HIV testing and if HIV positive, CD4 cell count (FACScount®, Becton Dickinson, San Jose, CA). HIV infection was defined as a positive test result using standard ELISA tests in parallel (Enzygnost anti HIV-1/HIV-2 Plus®; Dade Behring Diagnostics, Marburg, Germany, and Vironostika HIV Uni-Form II Ag/Ab® Biomerieux, Boxtel, The Netherlands) and a third ELISA test (BioRad HIV-1/2 Plus O EIA®, Bio-Rad Laboratories, Redmond, WA) [11]. TB and HIV clinical information was abstracted from the TB clinic surveillance registers, from which HIV-status (determined by rapid test according to the national guidelines) was used if ELISA results were unavailable. HIV-infected patients were offered HIV-care and treatment according to national policy.

### Data Management

Responses to the questionnaires were collected on paper forms and double entered; discrepancies were resolved from the original forms. Records were linked with HDSS data, to acquire geographic coordinates to determine the absolute distance between residencies and health facilities, and to obtain a socio-economic ranking score (SES). The SES ranks households socio-economic position based on multiple component analysis of data on the main source of income of the household head, source and treatment of drinking water, cooking fuel used, and ownership of cattle, a radio, television, bicycle, sofa, and lantern [19], [20]. The duration of cough in the actively detected patients was obtained from interviews at the time of the survey to avoid recall bias due to variable time between survey enrolment and care-seeking interview.

### Statistical Analysis

To explore factors that were possibly associated with the type of case detection, the dependent variable in the statistical analysis indicated whether the patient was detected through passive case detection, or not which implied that the patient was found through active case detection. Therefore, in the results an OR>1 represents relatively good or faster, and an OR<1 relatively poor or slower detection through passive case finding. The study had 80% power, at a two-sided significance level of 0.05, to detect a risk factor associated with passive case finding with an OR 0.47 and a prevalence of 40% among passively detected cases [21]. In univariable analysis the chi-square test, Fisher’s exact test, student t-test and Wilcoxon rank sum test were used where appropriate. In logistic regression, HIV, gender and age were added first and maintained in the model. Other risk factors were considered based on plausibility, a p-value <0.25 for the univariable association with the outcome, or a p-value <0.10 for the interaction with HIV. We first examined HIV and socio-demographic risk factors only, followed by a model where clinical factors that likely increased the probability of a TB diagnosis (duration of cough, sputum smear result and the ability to work normally) were also included. We continued the analysis in HIV-infected and uninfected patients separately because of interactions. We repeated the analysis among persons with smear-positive TB only, to exclude the influence of different case definitions for smear-negative TB, and restricted to patients who reported a cough for >14 days, since prolonged cough is generally recognized as a symptom that should prompt TB investigations [17]. Missing values for explanatory variables (shown in Table S1) were multiply imputed [22]. Prior to starting the multivariable analysis 10 multiple imputed data sets were created (using the SAS proc mi command) that included all the variables that were considered for the analysis model. Parameter estimates from each imputed data set were then combined to get a final single set of parameter estimates.

To further explore the contribution of patient and provider related factors to case detection, we examined as a secondary objective factors associated with contacting a public health provider among the actively detected patients, in univariable analysis and stratified by HIV-status. We used SAS 9.2 (SAS Institute Inc., Cary, North Carolina, USA) for data analysis.

### Validation

Among the actively detected patients, we compared the responses to questions on smoking during the household interviews with the responses during the later interviews at the clinics. The response ‘never smoked’ was repeated by 40 (95%) of 42 participants who reported ‘never smoked’ in the household interview. Of 23 people who reported ‘current smoking’ in the household interviews this was repeated by 4/23 (17%) while 16 (70%) reported ‘past smoking’ in the clinic interviews. We assumed a similar low repeatability for the question on alcohol consumptions and combined current and past consumption both for smoking and alcohol intake.

## Results

**Figure 1:**
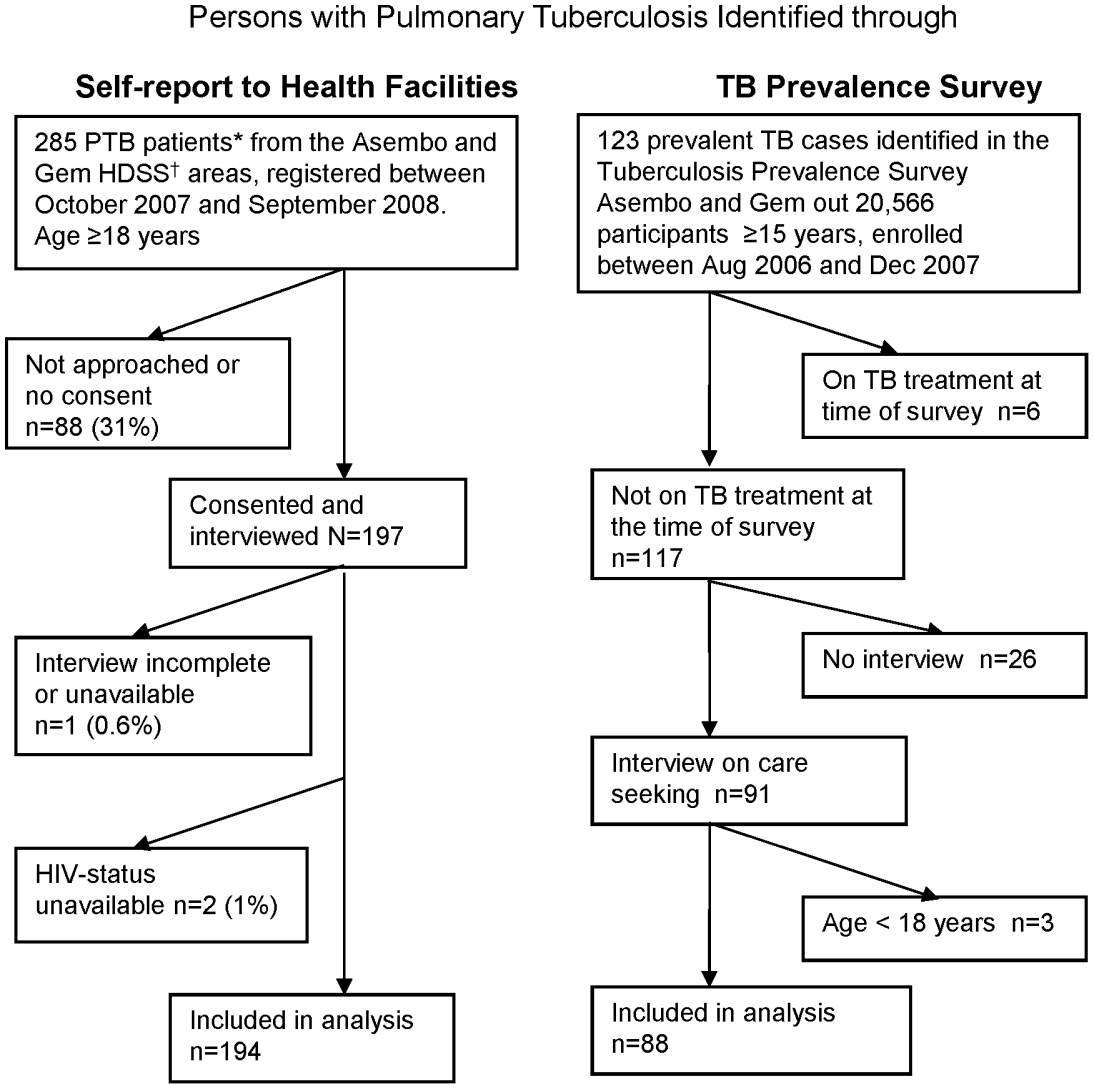
Selection of TB patients (N = 282) included in the analysis. Footnote to Figure1: PTB = Pulmonary Tuberculosis *PTB patients classified as new by the TB clinics, i.e. who did not receive TB treatment in the last 2 years. †HDSS = Health and Demographic Surveillance System. The Asembo and Gem areas are included in the HDSS since 2002 and the Karemo area since 2007. Patients from Karemo are not included in this analysis.

Of the 282 PTB patients included in the analysis (Figure 1), 194 were identified through passive case detection and met the inclusion criteria for this analysis. These 194 persons comprised 68% of 285 PTB patients who were registered at the TB-clinics in the area during the same period and would have been eligible. The enrolled patients had similar distributions of age, gender, HIV- and smear-status as those not in the study, but the latter had more missing data for HIV- and smear-status (data not shown). The 88 patients identified through active case detection and eligible for this analysis comprised 77% of the prevalent patients identified in the survey who were 18 years or older. They did not differ significantly by age, gender, HIV-status (if known), smoking, education, migration status or presence of cough of any duration from those without interview (data not shown). Interviewed prevalent patients were more often smear-positive (43/88 (49%) compared to 5/25 (20%) of those without interview, p = 0.01), and more often reported a cough for at least 2 weeks (52/88 (59%) versus 7/25 (28%), p = 0.02).

The passively detected patients were younger (median age 32 years; inter-quartile range (IQR) 25–43) than the actively detected patients (40 years; IQR 28–58, p = 0.0002). Other demographic characteristics were similar in both groups; 149 (53%) were female, 278 (99%) of Luo ethnicity, 148 (53%) were married, 187 (66%) had (some) primary education, 87 (31%) lived from subsistence farming and 67 (24%) did not have an independent income (Table S1).

HIV-infection was confirmed in 152 (78%) passively detected and 45 (51%) actively detected patients (OR 3.5, 95% CI 2.0–5.9). The median CD4 cell count was similar among passively detected (168/µl, IQR 81–319) and actively detected patients (205/µ, IQR 143–297; p = 0.25). All passively detected patients reported symptoms, and 138 (91%) HIV-infected and 35 (83%) HIV-uninfected reported a cough (p = 0.17). Of the actively detected patients 43 (96%) HIV-infected and 26 (60%) HIV-uninfected reported a cough (p<0.001), while 10 (11%) reported other symptoms and 9 (10%) none. In patients reporting a cough, the median duration was 4.3 months (IQR 1.6–10.2) in passively detected and 0.7 months (IQR 0.5–1.8; p = 0.017) in actively detected patients, and did not differ by HIV-status. Among smear-positive passively detected patients, the median duration of cough was longer in HIV-uninfected (6.9 months, IQR 4.8–10.1) than in HIV-infected patients (4.0 months, IQR 1.8–10.9; p = 0.05). At the time of PTB diagnosis, HIV-infected passively detected patients were relatively more ill by their self-report, while HIV-uninfected actively detected patients were relatively less ill (Tables 1,2).

**Table 1:** Risk factors associated with the probability of passive case detection among 197 HIV-infected pulmonary TB patients.

		Passively Detected		Actively Detected		Crude OR		Adjusted* OR	
		n	(%)	n	(%)	(95% CI)		(95% CI)	
Total		152		45					
Sex									
Female		87	(57)	23	(51)	1.3	(0.66–2.5)	0.91	(0.40–2.0)
Male		65	(43)	22	(49)	1		1	
Age categories									
18–34		86	(57)	22	(49)	1			
35–54		52	(34)	19	(42)	0.70	(0.35–1.4)		
55+		14	(9)	4	(9)	0.90	(0.27–3.0)		
								
Age - median [years] (IQR)		31(26–41)		36 (28–44)		p = 0.29			
OR per 10 years increase in age						0.87	(0.66–1.2)	0.91	(0.68–1.2)
Alcohol use									
Never		90	(60)	20	(49)	1		1	
Current or past use		60	(40)	21	(51)	0.64	(0.32–1.3)	0.56	(0.24–1.3)
missing		2		4					
Smoking									
Never smoked		121	(80)	31	(69)	1			
Current or past smoking		30	(20)	14	(31)	0.55	(0.26–1.2)		
missing		1							
Type of PTB by Sputum smear									
negative		82	(60)	20	(44)	1.9	(0.94–3.7)		
positive		55	(40)	25	(56)	1			
not done/missing		15							
Reported cough									
Cough of any duration		138	(91)	43	(96)	p = 0.53			
Duration in all patients with cough									
median (IQR) months		3.7 (1.6–10.9)		0.7(0.5–1.8) p<0.001					
per 1 month increase						1.5	(1.2–1.8)		
Duration in smear+ patients with cough									
median (IQR) CD4 cell count		4.0 (1.8–10.9)		0.9 (0.5–1.8) p<0.001					
median [cells/µl] (IQR)		168.0 (80.9–318.5)		205.5 (142.5–296.7) p = 0.25					
Missing		1		1					
Able to work at time of diagnosis/survey									
Normal		18	(12)	10	(23)	1			
Light work		58	(39)	21	(48)	1.5	(0.61–3.9)		
Unable		73	(49)	13	(30)	3.1	(1.2–8.3)		
Missing		3		1					
Able to walk at time of diagnosis/survey									
Yes		116	(78)	41	(93)	1			
No		35	(23)	3	(7)	4.1	(1.2–14)		
Missing									

OR = odds ratio CI = confidence interval sd = standard deviation.

* adjusted for HIV, age, sex and use of alcohol. Only HIV and socio-demographic factors were considered in the model.

**Table 2:** Risk factors associated with the probability of passive case detection among 85 HIV-uninfected pulmonaryTB patients.

		Passively Detected		Actively Detected			Crude OR		Adjusted* OR	
		n	(%)	n	(%)		(95% CI)		(95% CI)	
Total		42		43						
Sex										
Female		14	(33)	25	(58)		0.36	(0.15–0.87)	0.27	(0.10–0.73)
Male		28	(67)	18	(42)		1		1	
Age categories (years)										
18–34		19	(45)	9	(21)		1			
35–54		12	(29)	12	(28)		0.47	(0.15–1.5)		
55+		12	(29)	22	(51)		0.26	(0.09–0.75)		
Age - median [years] (IQR)		36.5(23–55)		55 (37–72) p = 0.004						
OR per 10 years increase in age							0.73	(0.58–0.91)	0.76	(0.60–0.97)
Alcohol use										
Never		23	(55)	10	(26)		1		1	
Current or past use		19	(45)	28	(74)		0.30	(0.12–0.76)	0.37	(0.13–1.0)
missing				5						
Smoking										
Never smoked		28	(67)	22	(51)		1			
Current or past smoking missing		14	(33)	21	(49)		0.52	(0.22–1.3)		
Type of PTB by Sputum smear										
negative		13	(34)	25	(58)		0.37	(0.15–0.92)		
positive		25	(66)	18	(42)		1			
not done/missing		4								
Reported cough										
Cough of any duration		35	(83)	26 (60) p = 0.02						
Duration in all patients with cough median (IQR)		5.2 (3.6–10.1)		0.9 (0.3–1.8) p<0.001						
per 1 month increase							1.5	(1.2–1.8)		
Duration in smear+ patients with cough median (IQR)		6.9 (4.8–10.1)		0.9 (0.4–1.8) p<0.001						
Able to work at time of diagnosis/survey										
Normal		12	(30)	26		(60)	1			
Light work		12	(30)	14		(33)	1.9	(0.66–5.2)		
Unable		16	(40)	3		(7)	12	(2.8–47)		
Missing		2								
Able to walk at time of diagnosis/survey										
Yes		35	(83)	41		(95)	1			
No		7	(17)	2		(5)	4.1	(0.80–21)		

OR = odds ratio CI = confidence interval sd = standard deviation.

* adjusted for HIV, age, sex and use of alcohol. Only HIV and socio-demographic factors were considered in the model.

When adjusted for HIV-status and socio-demographic factors, the probability of passive case detection was reduced in women (aOR 0.54, 95% CI 0.29–0.99), in patients who were older (aOR 0.82, 95% CI 0.69–0.98 per 10 years increase), or reporting current or past use of alcohol (aOR 0.42, 95% CI 0.23–0.79). HIV increased the probability of detection through the passive approach (aOR 2.7, 95% CI 1.5–4.8), but less so when adjusted for duration of cough, ability to work and smear-status (aOR 1.8, 95% CI 0.85–3.7). HIV had no effect when restricted to smear-positive patients only (aOR 0.94 (95% CI 0.29–3.0). In HIV-uninfected patients, the associations with female sex (aOR 0.27 (95% CI 0.10–0.73) and increasing age (aOR 0.76, 95% (CI 0.60–0.97) were stronger, and a longer duration of cough and impaired ability to work were more strongly associated with passive case detection (Table 2). In HIV-infected patients these associations were weaker and not significant (Table 1). A diagnosis of smear-negative PTB was associated with passive case detection (aOR 2.6, 95%CI 1.1–5.8); this association was stronger when the analysis was restricted to patients reporting a cough for >14 days (Table 3).

**Table 3:** Factors associated with the probability of passive case detection by HIV-status, adjusted for cough, ability to work and smear-status in all PTB patients (n = 282), smear-positive patients only (n = 123), and patients reporting a cough for more than 2 weeks (n = 198).

Odds Ratio’s	Crude	Adjusted	(95%CI)	Crude	Adjusted	(95%CI)	Crude	Adjusted	(95%CI)
	HIV-infected PTB patients								
	All (n = 197)			Smear-positive (n = 80)			Reporting Cough >2 weeks (n = 151)		
Gender									
Female	1.3	0.74	(0.31–1.8)	1.0	0.63	(0.16–2.4)	1.6	1.1	(0.36–3.5)
Male	1	1		1	1		1	1	
Age per 10 years increase	0.87	0.93	(0.67–1.3)	0.74	0.78	(0.46–1.4)	0.78	0.75	(0.49–1.15)
Alcohol use									
Never	1	1		1	1		1	1	
Current or past use	0.64	0.38	(0.15–1.0)	0.88	0.40	(0.10–1.6)	0.60	0.48	(0.15–1.6)
Cough per 1 month increase	1.5	1.4	(1.1–1.6)	1.3	1.4	(1.1–1.8)	1.2	1.3	(1.1–1.6)
Able to work at diagnosis (survey)									
Normal	1	1		1	1		1	1	
Light work	1.5	1.1	(0.40–3.1)	1.0	0.49	(0.08–3.1)	1.4	0.77	(0.19–3.2)
Unable	3.1	3.3	(1.1–10)	2.6	2.2	(0.37–13)	3.0	2.7	(0.62–12)
Type of PTB by Sputum smear									
Negative	1.9	2.5	(1.1–5.8)				3.9	4.6	(1.5–14)
Positive	1	1					1	1	
	**HIV-uninfected PTB patients**								
	**All (n = 85)**			**Smear-positive (n = 43)**			**Reporting Cough >2 weeks (n = 47)**		
Gender									
Female	0.36	0.32	(0.08–1.3)	0.20	0.22	(0.02–2.9)	0.52	0.20	(0.01–2.9)
Male	1	1		1	1		1	1	
Age per 10 years increase	0.73	0.65	(0.45–0.95)	0.68	0.55	(0.28–1.1)	0.75	0.70	(0.38–1.3)
Alcohol use									
Never	1	1		1	1		1	1	
Current or past use	0.30	0.42	(0.10–1.8)	0.26	0.48	(0.04–5.7)	0.23	0.05	(0.00–1.1)
Cough per 1 month increase	1.5	1.4	(1.2–1.7)	1.8	1.8	(1.1–3.0)	1.4	1.5	(1.1–2.1)
Able to work at diagnosis (survey)									
Normal	1	1		1	1		1	1	
Light work	1.9	4.0	(0.88–18)	1.6	1.4	(0.1–16)	1.4	5.0	(0.4–59)
Unable	12	35	(4.7–259)	13	35	(0.9–1305)	12	35	(4.7–259)
Type of PTB by Sputum smear									
Negative	0.37	0.99	(0.19–5.1)				1.1	0.99	(0.08–12.7)
Positive	1	1					1	1	

CI = Confidence interval PTB = pulmonary tuberculosis.

Missing variables (see table 1 and 2) were multiply imputed before logistic regression.

* Not all effects reach statistical significance in the small group of smear-positives, but the full model is shown to show trends.

Reported smoking (current or past) was only associated with poorer passive case finding in univariable analysis (OR 0.45, 95% CI 0.26–0.77) and was collinear with alcohol use. Education level, source of income, and socio-economic wealth ranking score were considered in multivariable analysis, but not associated with method of case-detection. Living at <1 and ≥5 km of a TB diagnostic facility increased case-finding through passive detection in univariable analysis, but not when adjusted for other variables (Table S1).

### Provider Contact

Of the 88 patients identified through active case finding, 25 (28%) had not consulted any formal or informal health provider at the time of survey. Of those 25, ten (40%) had reported a cough for >14 days and 4/25 (16%) were asymptomatic. Thirty-nine of the 88 patients (44%) reported at least one care-seeking effort during the 6 months prior to their participation in the survey, of whom 13/39 (33%) had consulted a private clinic, pharmacy, herbalist or a community health volunteer only, and 26 (67%) a public facility (hospital, health centre or dispensary). The latter 26/39 patients were younger (mean age 38.8 yrs; sd 18.9 versus 47.7 yr; sd 17.7, p = 0.04) and 18/26 (62%) were smear-positive at the time of the survey versus 25/62 (40%) of the patients who had not consulted a public facility. They did not significantly differ in gender, HIV-status, reported alcohol use, duration of cough or inability to work (data not shown). Of the passively detected patients 151 (78%) had consulted more than one care provider.

## Discussion

This comparison between PTB patients found through passive case detection and patients found actively through a prevalence survey showed that passive case detection resulted especially among HIV-uninfected patients in less adequate case detection among women, and persons who were older, while reported use of alcohol decreased case detection in both HIV-infected and uninfected patients.

The effect of HIV on case detection in this study should be interpreted as faster rather than better detection of HIV-infected TB patients through passive case detection. Since we modeled the odds with passively detected patients in the numerator, the ORs may be interpreted similarly to ratios of the patient diagnostic rate (PDR) [23]. The PDR has the notification rate in the numerator and the prevalence rate in the denominator and is an indicator of the relative rate at which TB cases are detected. In our earlier report we compared the prevalence estimate from the prevalence survey with provincial notification data. We estimated that the rate at which new PTB cases were detected was higher for the HIV-infected, while the proportion of HIV-infected PTB cases detected was lower (56%) than that of the HIV-uninfected (65%) [11], explained by a more rapid progression to severe disease or death in HIV-infected TB patients [3], [24], [25]. The presence of symptoms and illness partly explain the faster detection of HIV-infected patients. As expected [5]–[7], [9], the probability of passive detection increased with longer duration of cough and increased illness, which likely prompts care-seeking by patients and TB diagnosis by health workers [26], [27].

As a consequence of rapid disease progression, a shorter duration of cough in HIV-infected patients would be expected, which we only found in smear-positive patients, in whom bacteriologically active PTB is more likely. Passively detected patients with clinically diagnosed smear-negative PTB likely include false positive diagnoses due to the low specificity of CXR [28] and clinical signs, especially in HIV-infected persons [29]. While due to the low sensitivity of clinical diagnosis, patients with a shorter durations may have been missed [28], [30]. Similar durations of cough in passively detected patients, regardless of HIV-status may also be due to imprecise recall of the duration of symptoms, or a higher prevalence of symptoms in HIV-infected persons in general, i.e., not attributable to TB [31]. In actively detected patients the duration of cough may be a relative underestimate, since it was obtained when the TB diagnosis was still unknown.

Slower case detection in women and the elderly is consistent with reports on diagnostic delay from other African [3], [8], [32]–[36] and Asian populations [8], [24] with low HIV-prevalence. This contrasts with a shorter duration of disease in the elderly [37], perhaps attributable to increased mortality. Among the patients identified through active case finding, provider consultation prior to the prevalence survey was also lower among the elderly patients, but did not differ by sex. Less knowledge [38] or resources and opportunity to attend a health facility may be contributing factors among the elderly. In women lower smear-positivity may contribute to slower case detection [39]. The analysis on provider consultation among actively detected patients is however limited by small numbers. Several studies reported alcoholism or substance abuse as a risk factor for diagnostic delay^3^. In this study the use of alcohol and smoking were rather crude and collinear indicators, and may be indicative of social factors rather than a causal effect. We did not find an effect of socio-economic position or education level on case detection, which is unexpected since TB was more prevalent in households with a lower socio-economic position in our own and other prevalence surveys [11], [40]. The lack of an effect between case detection and SES ranking in this study may also be because within this rather poor population the absolute differences in wealth are too small to show an effect, or due to low explanatory power of the assets included in the score, or to missing values caused by the inability to link some patients with the HDSS database.

Overall, the power of this study was limited by the number of prevalent cases, and by different case definitions for smear-negative TB discussed above. However, the analysis restricted to smear-positive cases only, showed similar trends in risk factors. Due to partial overlap in enrolment period of the two groups, some patients who were identified through the prevalence survey might otherwise have been identified through self-report at a later stage. We expect the bias from this effect to be small since only 38% of the villages were included in the prevalence survey, and the majority of the self-reported cases from these villages were diagnosed 6–24 months after their village participated in the prevalence survey. If passive case detection improved in general due to publicity around the survey, this would decrease the differences between the passively and actively detected cases, and thus not invalidate the results.

Referral of patients attending HIV-clinics may have increased the numbers of HIV-infected and smear-negative self-reported patients. However, at the time of study intensified TB case finding in HIV-clinics was not routine. Excluding patients who reported knowledge of their HIV-status prior to TB diagnosis only marginally reduced the observed associations (data not shown).

The population in the prevalence survey had fewer males and younger persons compared to the composition according to the HDSS [11]. Bias could occur if males and younger persons who are absent during surveys due to labour related migration would return to their home area when ill [41]. However, only 5% of the TB patient reported recent relocation to the area because of illness. Moreover, the prevalent patients included in this study had similar distributions of age and gender as survey participants (n = 85) who reported to already be on TB treatment at the time of survey. Over-representation of sick people in the survey (to obtain free health care), would likely make the actively detected patients more similar to those found passively, and thus not invalidate our results.

This study shows the importance of differentiating TB case finding strategies by HIV-status. Early diagnosis of HIV-infection allows for intensified TB case finding as well as isoniazid preventive therapy and early initiation of ART [42], all contributing to lowering TB disease burden [37], [43], [44]. In HIV-uninfected persons, improving TB case finding will require increased suspicion of TB in persons who are less ill, suggesting a role for active case finding [6], [45], [46]. A mobile active case finding intervention showed high participation of women in Harare [47], and would be worthwhile piloting in a rural area as well. The large numbers of HIV-attributable TB may reduce TB suspicion in HIV-uninfected patients with milder illness. And moreover mask case detection patterns in HIV-uninfected patients, who are a minority. Projects piloting novel case finding approaches should consider this, and separate monitoring of case detection by HIV-status would be useful.

In conclusion, in HIV-uninfected TB patients, case detection through passive was associated with inferior detection of women, persons who were older, and regardless of HIV-status, patients who reported use of alcohol. In addition to targeting all HIV-infected for intensified TB case finding, additional efforts are required to improve case detection in HIV-uninfected risk groups.

## Supporting Information

Table S1 **Characteristics of 282 PTB cases included in the analysis.**
(DOC)
