# Supplementary material for: Risk Factors for Inadequate TB Case Finding in Rural Western Kenya: A Comparison of Actively and Passively Identified TB Patients
Source: PLoS One. 2013 Apr 25;8(4):e61162. doi: 10.1371/journal.pone.0061162 (PMC3636249; doi:10.1371/journal.pone.0061162)
Supplement: Table S1 — Characteristics of 282 PTB cases included in the analysis. (DOC) [file pone.0061162.s001.doc]

| **Table S1. Characteristics of 282 PTB cases included in the analysis** | | | | | | | | |
| --- | --- | --- | --- | --- | --- | --- | --- | --- |
|  |  | **Passive case detection** | | **Active case detection** | | Crude OR (95% CI) | | |
|  |  | n | % | n | % |  |  |  |
| Total |  | 194 |  | 88 |  |  |  |  |
| Gender |  |  |  |  |  |  |  |  |
|  | Female | 101 | 52% | 48 | 55% | 0.91 | 0.55 | 1.50 |
|  | Male | 93 | 48% | 40 | 45% | 1 |  |  |
| Age - categories |  |  |  |  |  |  |  |  |
|  | 18-34 | 105 | 54% | 31 | 35% | 1 |  |  |
|  | 35-54 | 64 | 33% | 31 | 35% | 0.61 | 0.34 | 1.10 |
|  | 55+ | 25 | 13% | 26 | 30% | 0.28 | 0.14 | 0.56 |
|  |  |  |  |  |  |  |  |  |
| Age - mean (years)/sd | | 36.0 | sd 14.2 | 45.1 sd 18.4 | |  |  |  |
| OR per 10 years increase in age | |  |  |  |  | 0.71 | 0.61 | 0.83 |
| Type of PTB by Sputum smear | |  |  |  |  |  |  |  |
|  | Negative | 95 | 49% | 45 | 51% | 1.14 | 0.68 | 1.90 |
|  | Positive | 80 | 41% | 43 | 49% | 1 |  |  |
| Not done / missing | | 19 | 10% |  |  |  |  |  |
| HIV status |  |  |  |  |  |  |  |  |
|  | Positive | 152 | 78% | 45 | 51% | 3.46 | 2.02 | 5.93 |
|  | Negative | 42 | 22% | 43 | 49% | 1 |  |  |
| Smoking |  |  |  |  |  |  |  |  |
|  | Never smoked | 149 | 77% | 46 | 58% | 1 |  |  |
|  | Past smoking | 42 | 22% | 29 | 37% | 0.45 | 0.25 | 0.80 |
|  | Current smoking | 2 | 1% | 4 | 5% | 0.15 | 0.03 | 0.87 |
|  | missing | 1 |  | 9 |  |  |  |  |
| Alcohol use |  |  |  |  |  |  |  |  |
|  | Never | 113 | 58% | 30 | 38% | 1 |  |  |
| Used to but ceased | | 70 | 36% | 38 | 48% | 0.49 | 0.28 | 0.86 |
|  | Current use | 9 | 5% | 11 | 14% | 0.22 | 0.08 | 0.57 |
|  | missing | 2 |  | 9 |  |  |  |  |
| Education level |  |  |  |  |  |  |  |  |
|  | none | 22 | 11% | 14 | 16% | 1 |  |  |
|  | (some) primary | 127 | 65% | 60 | 68% | 1.35 | 0.65 | 2.82 |
| (some) sec or post-sec | | 44 | 23% | 14 | 16% | 2.00 | 0.81 | 4.92 |
|  | missing | 1 |  |  |  |  |  |  |
| Income Source |  |  |  |  |  |  |  |  |
| No independent income | | 48 | 25% | 19 | 22% | 1.6 | 0.82 | 3.2 |
| Subsistance farming | | 53 | 27% | 34 | 39% | 1 |  |  |
| Other Independent income source | | 93 | 48% | 35 | 40% | 1.7 | 0.95 | 3.0 |
| Socio-economic score | |  |  |  |  |  |  |  |
|  | Lowest - 1 | 24 | 12% | 18 | 24% | 0.80 | 0.33 | 1.9 |
|  | 2 | 26 | 13% | 14 | 16% | 1.11 | 0.45 | 2.8 |
|  | 3 | 33 | 17% | 9 | 10% | 2.2 | 0.83 | 5.8 |
|  | 4 | 23 | 12% | 19 | 22% | 0.73 | 0.30 | 1.8 |
|  | Highest - 5 | 25 | 13% | 15 | 20% | 1 |  |  |
|  | missing | 63 | 32% | 13 |  |  |  |  |
| Marital status |  |  |  |  |  |  |  |  |
|  | Single | 28 | 14% | 9 | 10% | 1 |  |  |
|  | Married | 100 | 52% | 48 | 55% | 1.5 | 0.65 | 3.4 |
|  | Widowed | 42 | 22% | 22 | 25% | 0.92 | 0.49 | 1.7 |
|  | Separated | 22 | 11% | 9 | 10% | 1.2 | 0.50 | 2.7 |
|  | Other | 2 | 1% | 0 | 0% | - | - | - |
| Ethnicity |  |  |  |  |  |  |  |  |
|  | Luo | 191 | 98% | 87 | 99% | 1 |  |  |
|  | Other | 3 | 2% | 1 | 1% | 1.37 | 0.14 | 13.32 |
| Distance to TB Diagnostic Facility | |  |  |  |  |  |  |  |
|  | 0 - <1km | 20 | 10% | 2 | 2% | 6.78 | 1.50 | 30.63 |
|  | 1 - <3km | 59 | 30% | 40 | 45% | 1 |  |  |
|  | 3 - <5km | 68 | 35% | 33 | 38% | 1.40 | 0.78 | 2.49 |
|  | 5+ km | 44 | 23% | 13 | 15% | 2.30 | 1.10 | 4.80 |
|  | missing† | 3 | 2% |  |  |  |  |  |
| Reported duration of Cough | |  |  |  |  |  |  |  |
|  | No cough | 21 | 11% | 19 | 22% | 2.6 | 1.1 | 6.5 |
|  | up to 14 days | 13 | 7% | 31 | 35% | 1 |  |  |
|  | > 2 wks - 8 wks | 34 | 18% | 24 | 27% | 3.4 | 1.5 | 7.8 |
|  | > 8 wks - 6 months | 60 | 31% | 10 | 11% | 14 | 5.6 | 36 |
|  | > 6 months | 66 | 34% | 4 | 5% | 39 | 12 | 130 |
|  |  |  |  |  |  |  |  |  |
| Median duration (months), (IQR) | | 4.3 (1.6-10.2) | | 0.7 (0.5-1.8) | |  |  |  |
| Cough duration- per 1 month increase | |  |  |  |  | 1.4 | 1.2 | 1.6 |
| Able to work at time of diagnosis (survey) | |  |  |  |  |  |  |  |
|  | Normal | 30 | 15% | 36 | 41% | 1 |  |  |
|  | Light work | 70 | 36% | 35 | 40% | 2.4 | 1.3 | 4.5 |
|  | Unable | 89 | 46% | 16 | 18% | 6.7 | 3.3 | 13.7 |
|  | DNK or missing | 5 | 3% | 1 |  |  |  |  |
| OR= odds ratio; DNK=does not know; PTB=pulmonary tuberculosis; IQR=interquartile range | | | | | | | | |
